# Positive Modulatory Interactions of NMDA Receptor GluN1/2B Ligand Binding Domains Attenuate Antagonists Activity

**DOI:** 10.3389/fphar.2017.00229

**Published:** 2017-05-09

**Authors:** Douglas Bledsoe, Ceyhun Tamer, Ivana Mesic, Christian Madry, Bradley G. Klein, Bodo Laube, Blaise M. Costa

**Affiliations:** ^1^Edward Via College of Osteopathic MedicineBlacksburg, VA, USA; ^2^Department of Neurophysiology and Neurosensory Systems, Technische Universität DarmstadtDarmstadt, Germany; ^3^Department of Neuroscience, Physiology and Pharmacology, University College LondonLondon, UK; ^4^Department of Biomedical Sciences & Pathobiology, Virginia-Maryland College of Veterinary Medicine, Virginia TechBlacksburg, VA, USA; ^5^School of Neuroscience, Virginia TechBlacksburg, VA, USA

**Keywords:** NMDA receptor, Ligand Binding Domain (LBD), competitive antagonists, memantine, interface

## Abstract

N-methyl D-aspartate receptors (NMDAR) play crucial role in normal brain function and pathogenesis of neurodegenerative and psychiatric disorders. Functional tetra-heteromeric NMDAR contains two obligatory GluN1 subunits and two identical or different non-GluN1 subunits that include six different gene products; four GluN2 (A–D) and two GluN3 (A–B) subunits. The heterogeneity of subunit combination facilities the distinct function of NMDARs. All GluN subunits contain an extracellular N-terminal Domain (NTD) and ligand binding domain (LBD), transmembrane domain (TMD) and an intracellular C-terminal domain (CTD). Interaction between the GluN1 and co-assembling GluN2/3 subunits through the LBD has been proven crucial for defining receptor deactivation mechanisms that are unique for each combination of NMDAR. Modulating the LBD interactions has great therapeutic potential. In the present work, by amino acid point mutations and electrophysiology techniques, we have studied the role of LBD interactions in determining the effect of well-characterized pharmacological agents including agonists, competitive antagonists, and allosteric modulators. The results reveal that agonists (glycine and glutamate) potency was altered based on mutant amino acid sidechain chemistry and/or mutation site. Most antagonists inhibited mutant receptors with higher potency; interestingly, clinically used NMDAR channel blocker memantine was about three-fold more potent on mutated receptors (N521A, N521D, and K531A) than wild type receptors. These results provide novel insights on the clinical pharmacology of memantine, which is used for the treatment of mild to moderate Alzheimer's disease. In addition, these findings demonstrate the central role of LBD interactions that can be exploited to develop novel NMDAR based therapeutics.

## Introduction

The N-methyl D-aspartate (NMDA) receptor is a subtype of the ionotropic glutamate receptor (iGluR) family. NMDA receptors have been implicated in the pathogenesis of several neurological and psychiatric disorders including Alzheimer's disease, epilepsy, amyotrophic lateral sclerosis, and schizophrenia (Monaghan and Jane, 2009). Like any other iGluR, NMDA receptors have four domains: clam shell shaped extracellular N-terminal domain (NTD) and ligand binding domain (LBD), an ion channel forming transmembrane domain (TMD), and an intracellular C-terminal domain (CTD). Functional NMDA receptors are composed of four subunits, expressed as either dihetero- or trihetero- forms with two obligatory glycine binding GluN1 subunits, and other two identical or different glutamate binding GluN2 subunits. There are four GluN2 (A-D) subunits each encoded by a separate gene. These four subunits have distinct physiological and pharmacological properties including spatiotemporal expression pattern, agonist potency, deactivation kinetics and intracellular signaling mechanisms. GluN1/2 subunit containing NMDA receptors are blocked by Mg^2+^ ions at resting membrane potential, and this blockade can be reversed by a depolarizing potential. Therefore, NMDA receptors are fully activated only during concurrent binding of agonists and depolarizing membrane potential, thus acting as co-incidence detectors. Additionally, there are two variants of GluN3 (A-B) subunits that can co-assemble with GluN1 to form excitatory glycine receptors (Chatterton et al., 2002; Madry et al., 2007; Smothers and Woodward, 2009) or GluN1/2/3 subunit containing triheteromeric NMDA receptors(Perez-Otano et al., 2016).

The central role of the LBD in NMDA receptor function has been demonstrated by numerous studies in the past two decades (Traynelis et al., 2010). The structural homology of this region with other subunits across the iGluR family has been exploited to develop a large number of competitive antagonists (Selfotel–Novartis, Gavestinel-GlaxoSmithKline, D-CPPene -Sandoz, and GV196771–GlaxoSmithKline) that serve as chemical tools to study NMDA receptor physiology or are considered as drug candidates for the treatment of neurological disorders and for preventing death and long term disability after stroke and traumatic brain injury in human beings, as extensively reviewed in (Muir, 2006) and (Traynelis et al., 2010). While the LBD acts as a promising drug target, high sequence similarity in that region impedes development of GluN2 subunit selective pharmacological agents which are of great clinical significance (Blaise et al., 2004; Kinarsky et al., 2005). The compounds binding at the LBD cleft are weakly selective to the GluN2 subunit of interest (Traynelis et al., 2010). Consequently, approaches to identify drug binding sites where amino acids are less conserved, and the development of compounds that target these binding sites, is great pharmaceutical interest; however, remained as a challenge. Discovery of a novel family of GluN2 specific compounds and their binding sites revealed the existence of a potential modulator-binding site at the GluN1/2 LBD dimer interface (Costa et al., 2010). Furthermore, the LBD dimer interface was predicted by molecular modeling as the primary binding site for a GluN1/2A selective potentiator (Kane and Costa, 2015). Recently, a number of high affinity compounds have been developed to positively modulate GluN1/2A receptor function, and some of these compounds have been already co-crystallized with GluN1/2A LBD constructs and were found to bind in the dimer interface (Hackos et al., 2016; Volgraf et al., 2016). These developments motivated us to further investigate the role of the LBD interface in the pharmacology of compounds that are known to bind outside of the LBD interface. Since the LBD interface modulates NMDAR function, agents that bind at the LBD interface may alter the activity of other drugs that bind elsewhere on the NMDAR complex. To test this hypothesis, we made point mutations at key residues that participate in stabilizing the dimer LBD interface and tested the activity of three classes of agents that act at other sites on the NMDAR complex.

**Figure 1:**
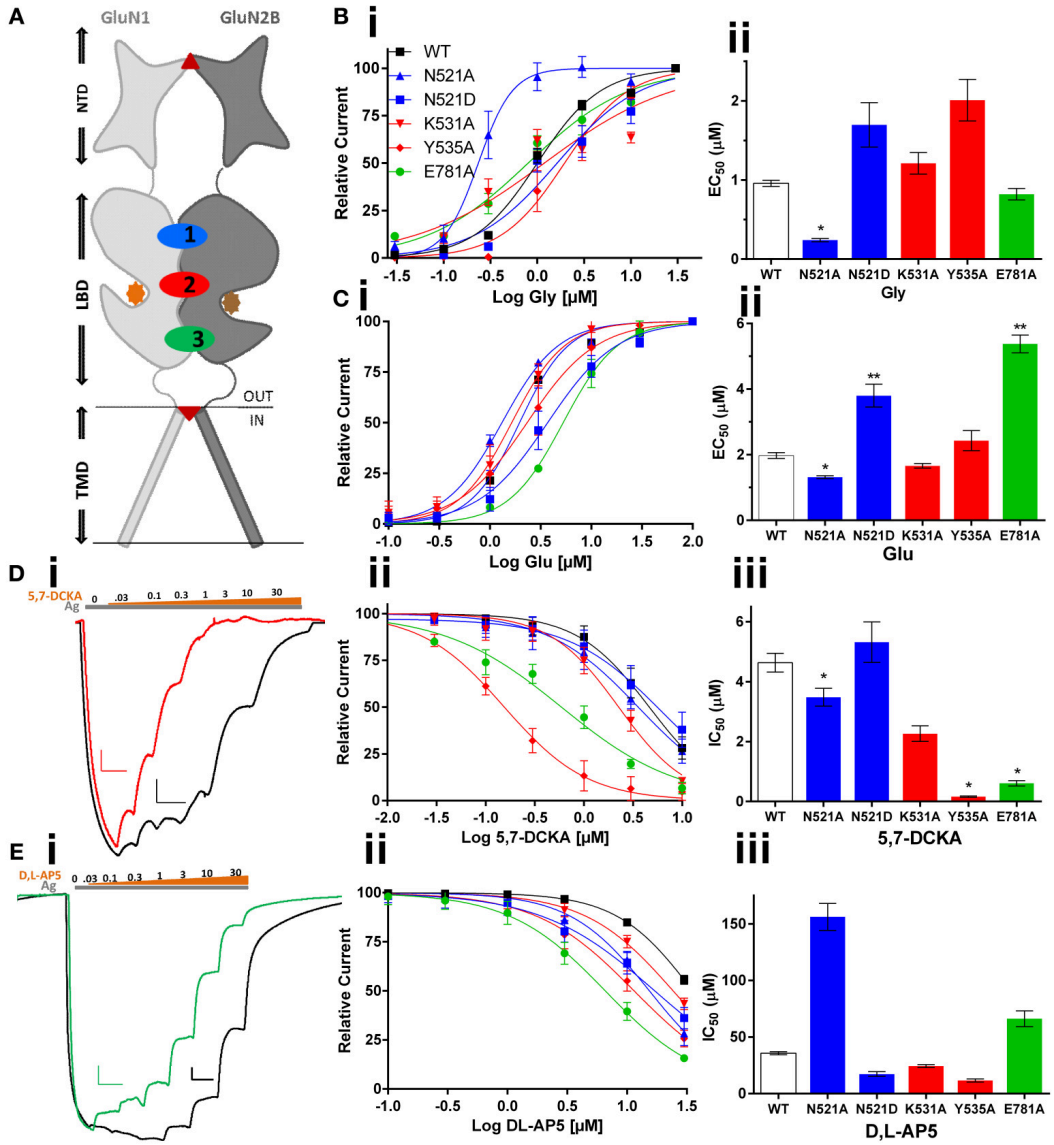
**Effect of GluN1 LBD mutations (co-expressed with GluN2B) on the activity of LBD cleft binding compounds. (A)** topology diagram shows the location of different domains (NTD, LBD, and TMD) in the GluN1 and GluN2B dimer. Glycine (orange circle) and glutamate (brown circle), ifenprodil (triangle), and memantine (inverted triangle) binding is marked. LBD interaction sites are labeled as 1 (Site-I, blue), 2 (Site-II, red), and 3 (Site-III, green). Following are the mutations made at these sites: N521A, N521D at site-I; K531A & Y535A at Site-II and E781A at Site-III. Mutation induced changes in potency of the co-agonist, glycine **(Bi,ii)** and agonist glutamate **(Ci,ii)** of the NMDA receptor. Traces represent dose response curves (black-wildtype, red-Y535A, green-E781A; gray and brown bar- agonist and antagonist, respectively) that show results obtained from glycine site antagonist, 5,7-DCKA (**Di–iii**, scale: X-axis 10 s, and Y-axis 25 nA) and the effect of glutamate site antagonist, DL-AP5 (**Ei–iii**, scale: X-axis 10 s, and Y-axis 100 nA). Statistical significance is marked as ^*^p < 0.01 or ^**^P < 0.001.

Systems biology has made remarkable contribution in the advancement of neuroscience research after the completion of human genome project. Particularly, the evolution of systems biology based mathematical modeling software programs incredibly improved our ability to analyze the x-ray crystallographic data and protein sequence, which are essential to identify novel drug binding sites. In the NMDA receptors, LBD interface encompasses a large (~35Å) chemical groove that is stabilized by intersubunit interactions at three different points, Site-I, II, and III (Furukawa et al., 2005; see Figure 1A). While the interaction between GluN1 and GluN2A subunits at these three points in GluN1/2A receptors is imperative, equivalent interactions in the other combinations of GluN2 (B, C, and D) subunit containing NMDA receptors have not been studied. Based on the comparison of amino acid sequences and three dimensional structures of full length NMDA receptor (Karakas and Furukawa, 2014), we have found that the interaction between GluN1 and co-assembling GluN2 is distinct for every subunit of the GluN2 family, despite sharing about 70–80% amino acid sequence identity at the LBD. Therefore, in the present study we have made point mutations in the site-I (N521A and N521D), site-II (K531A, Y535A) and site-III (E781A) of the GluN1 subunit LBD and co-expressed the mutants with wild type GluN2B subunits to study the role of domain stability in determining the pharmacology of compounds that are binding outside of the LBD interface.

## Materials and methods

Compounds: Compounds that are known to bind with NMDA receptors were obtained from either Tocris Bioscience, Bristol, UK (DL-AP5, cat# 76326-31-3; Memantine, cat # 19982-08-2; 5,7-DCKA cat#13112376-7), or Hellobio Ltd., Bristol, UK (Ifenprodil, cat# HB0339).

NMDA receptor constructs: cDNA encoding the NMDAR1a subunit (GluN1a) was a generous gift of Dr. Nakanishi (Kyoto, Japan). cDNA encoding the GluN2B (*pci_sepGluN2B*) was purchased from Addgene, Cambridge, MA. GluN1 mutants (N521A, N521D, K531A, Y535A, and E781A) were generated by site-directed mutagenesis (QuikChange XL site-directed mutagenesis kit; Stratagene, Amsterdam, The Netherlands) and confirmed by DNA sequencing. Plasmids were linearized with *NotI* (GluN1a wt and all five GluN1 mutants,) or *Xba1* (GluN2B), and transcribed *in vitro* with T7 (GluN1a, & GluN2B), SP6 (GluN1 mutants) RNA polymerase using the mMessage mMachine transcription kits (Ambion, Austin, TX, USA).

GluN subunit expression and electrophysiology in Xenopus oocytes: Stage IV frog oocytes were obtained from Xenopus-I, (Ann Arbor, MI, USA). NMDA receptor subunit cRNAs were dissolved in nuclease free sterile H_2_O. GluN1a and GluN2B cRNAs were mixed in a ratio of 1:1–3. 50 nL of the final cRNA mixture was microinjected (40–70 ng total) into the oocyte cytoplasm. Oocytes were incubated in ND-96 solution at 18°C prior to electrophysiological recordings (1–3 days). Electrophysiological responses were measured using a standard two-microelectrode voltage clamp [Warner Instruments (Hamden, Connecticut) model OC-725C] designed to provide fast clamp of large cells. The recording buffer contained 116 mM NaCl, 2 mM KCl, 0.3 mM BaCl2, and 5 mM HEPES, pH 7.4. Response magnitude was determined by the steady plateau response elicited by bath application of agonists (10 μM L-glutamate and 10 μM glycine) at a holding potential of −60 mV. Response amplitudes for functional NMDA receptors were generally between 0.1 and 2 μA. After obtaining a steady-state response to agonist application, agonist plus test compounds were applied, using 8-channel perfusion system (Automate Scientific, Berkeley, CA), on the oocytes and the responses were digitized for quantification (Digidata 1550A and pClamp-10, Molecular Devices, Sunnyvale, CA). Dose-response relationships were fit to a single-site with variable slope (GraphPad Prism, ISI Software, San Diego, CA, USA), using a non-linear regression to calculate IC_50_ or EC_50_ and percentage maximal inhibition. Statistical Analysis: Values given represent means (±) S.E. In order to present only highly significant results, statistical significance was determined at the alpha level *p* < 0.01 (^*^) and *p* < 0.001 (^**^) using a student's two-tailed, unpaired *t*-test.

## Effects of GluN1 mutations on compounds binding within the LBD cleft

The data obtained from the two electrode voltage clamp (TEVC) electrophysiology experiments reveal that GluN1/2B LBD interactions play a crucial role in determining potency of the ligands binding at the glycine or glutamate binding cleft of NMDA receptors. The topology diagram of the GluN1/2 dimer shows the location of the LBD, NTD, and TMD, and the three LBD interaction sites are numbered (Figure 1A). The site-I mutant, GluN1*(N521A)*, increased the potency of glycine (EC_50_: 0.96 vs. 0.24 μM, *p* < 0.01), glutamate (EC_50_:1.97 vs. 1.31 μM, *p* < 0.01) and 5,7-DCKA (IC_50_: 4.63 vs. 3.48 μM, *p* < 0.01), that is a glycine site competitive antagonist (Figures 1B–E, Table 1). However, the *N521A* mutant did not significantly affect the potency of glutamate site competitive antagonist DL-AP5 (IC_50_: wt, 35.67 vs. 156.1 μM; *p* > 0.01). Since the GluN1 *521*^st^ position is known to be crucial for intersubunit interactions(Furukawa et al., 2005), we have further studied the role of this position by generating another mutation (*N521D*) in such a way that side chain length will remain the same as wild type, but only the reactive groups will be changed. The amino (NH_2_) group of asparagine is replaced by the carboxylic acid (COO^−^) group of aspartic acid in the *N521D* mutant. In contrast to the *N521A* mutant, the *N521D* mutant decreased the glutamate potency (EC_50_: 1.97 vs. 3.49 μM; *p* < 0.01; Figure 1C). These findings reveal the influence of 521^st^ amino acid (Site-I) side chain in GluN1/2B interactions. Site-II mutations (*K531A* and *Y535A*) did not significantly affect the glycine or glutamate EC_50_. However, a remarkable more than 25-fold significant increase (IC_50_: 4.63 vs. 0.16 μM, *p* < 0.01) in 5,7-DCKA potency was observed with the *Y535A* mutant. Alternatively, DL-AP5 potency was not significantly changed by the site-II mutants. These results reveal that GluN1/2B intersubunit interactions through site-II largely influence the potency of competitive antagonist binding at GluN1, but have insignificant effect on glutamate site antagonist activity. The site-III mutation (*E781A*) was made close to the distal end of the GluN1/2B LBD interface, a region that is physically connected with the TMD and a more dynamic site of the LBD than the other two sites, as shown in Figure 1A. Despite the *E781A* mutation occurring at the GluN1 subunit, the potency of glutamate (that binds with GluN2) was significantly reduced (EC_50_:1.97 vs. 5.38 μM, *p* < 0.01). Converse to what was observed for glutamate, *E781A* increased the potency of the glycine site antagonist 5,7-DCKA (IC_50_:4.63 vs. 0.61 μM, *p* < 0.01), however, the potency of the glutamate antagonist, DL-AP5 was unaltered. This result corroborates the observations made with site I & II.

**Table 1:** **Summary of the effect of LBD mutations on NMDA receptor ligand pharmacology**.

**Ligand**	**GluN1**	**EC_50_ or IC_50_ (μM)**	**Correctness of fit (*R*^2)^**	**Hill Slope (nH)**	**N**	***P*-Value**
Glycine	WT	0.96 ± 0.04	0.985 ± 0.003	1.244 ± 0.084	5	–
	N521A	0.24 ± 0.02	0.983 ± 0.005	2.542 ± 0.156	6	0.0043^*^
	N521D	1.70 ± 0.28	0.958 ± 0.006	1.012 ± 0.055	4	0.0159
	K531A	1.21 ± 0.13	0.880 ± 0.004	0.630 ± 0.027	4	0.1429
	Y535A	2.01 ± 0.07	0.987 ± 0.003	1.329 ± 0.066	4	0.0159
	E781A	0.82 ± 0.07	0.975 ± 0.003	0.820 ± 0.036	6	0.3571
Glutamate	WT	1.97 ± 0.09	0.99 ± 0.0008	1.83 ± 0.072	7	–
	N521A	1.31 ± 0.04	0.992 ± 0.002	1.564 ± 0.061	4	0.0061^*^
	N521D	3.49 ± 0.32	0.994 ± 0.001	1.288 ± 0.051	9	0.0002^**^
	K531A	1.66 ± 0.07	0.992 ± 0.002	1.667 ± 0.158	8	0.0112
	Y535A	2.43 ± 0.31	0.991 ± 0.006	1.423 ± 0.124	8	0.0552
	E781A	5.38 ± 0.27	0.997 ± 0.001	1.651 ± 0.097	7	0.0006
5,7 DCKA	WT	4.63 ± 0.33	0.993 ± 0.002	−1.244 ± 0.089	7	–
	N521A	3.48 ± 0.32	0.992 ± 0.002	−1.061 ± 0.146	7	0.0025^*^
	N521D	5.32 ± 0.68	0.981 ± 0.005	−0.980 ± 0.113	6	0.7499
	K531A	1.66 ± 0.26	0.990 ± 0.005	−1.262 ± 0.131	4	0.0202
	Y535A	0.16 ± 0.02	1.000 ± 0.001	−1.061 ± 0.112	5	0.0016^*^
	E781A	0.61 ± 0.09	0.980 ± 0.003	−0.746 ± 0.038	4	0.004^*^
DL-AP5	WT	35.67 ± 1.30	0.999 ± 0.0001	−1.37 ± 0.095	4	–
	N521A	156.1 ± 11.91	0.994 ± 0.001	−1.318 ± 0.072	4	0.0286
	N521D	17.27 ± 2.08	0.987 ± 0.005	−0.932 ± 0.091	4	0.0286
	K531A	24.29 ± 1.30	0.998 ± 0.001	−1.195 ± 0.050	4	0.0286
	Y535A	11.59 ± 1.35	0.996 ± 0.002	−1.078 ± 0.047	4	0.0286
	E781A	66.16 ± 7.00	0.997 ± 0.001	−1.089 ± 0.072	4	0.0286
Zn^2+^	WT	2.73 ± 0.08	0.998 ± 0.0001	−1.038 ± 0.027	5	–
	N521A	4.99 ± 0.32	0.994 ± 0.002	−0.883 ± 0.050	4	0.0159
	N521D	5.70 ± 0.90	0.992 ± 0.004	−0.933 ± 0.053	4	0.0159
	K531A	6.25 ± 0.52	0.996 ± 0.003	−0.969 ± 0.058	4	0.0159
	Y535A	6.99 ± 1.32	0.960 ± 0.012	−0.858 ± 0.090	8	0.042
	E781A	3.06 ± 0.60	0.998 ± 0.0004	−1.020 ± 0.048	4	0.5635
Ifenprodil	WT	1.23 ± 0.36	0.977 ± 0.009	−0.698 ± 0.060	4	–
	N521A	8.70 ± 2.08	0.992 ± 0.002	−1.05 ± 0.090	6	0.0095^*^
	N521D	2.06 ± 0.50	0.958 ± 0.007	−0.679 ± 0.092	4	0.7714
	K531A	1.66 ± 0.87	0.952 ± 0.012	−0.725 ± 0.123	5	0.0286
	Y535A	2.34 ± 1.18	0.978 ± 0.002	−0.644 ± 0.099	4	0.7714
	E781A	1.52 ± 0.17	0.960 ± 0.013	0.146 ± 0.016	4	0.7714
Memantine	WT	1.43 ± 0.11	0.998 ± 0.0005	−0.953 ± 0.017	8	–
	N521A	0.64 ± 0.04	0.98 ± 0.006	−0.763 ± 0.015	6	0.0007^**^
	N521D	0.84 ± 0.10	0.9946 ± 0.0028	−0.907 ± 0.041	5	0.0093^*^
	K531A	0.60 ± 0.07	0.9966 ± 0.0010	−0.943 ± 0.009	5	0.0016^*^
	Y535A	1.58 ± 0.21	0.9816 ± 0.0055	−0.667 ± 0.020	6	0.6204
	E781A	0.87 ± 0.12	0.993 ± 0.0006	−0.77 ± 0.0078	4	0.0202

*Amino acid location is [site I (blue), II (red) and III (green)] color coded*.

* *p < 0.01*,

** *p < 0.001*.

## Effects of GluN1 mutations on non-competitive antagonists

**Figure 2:**
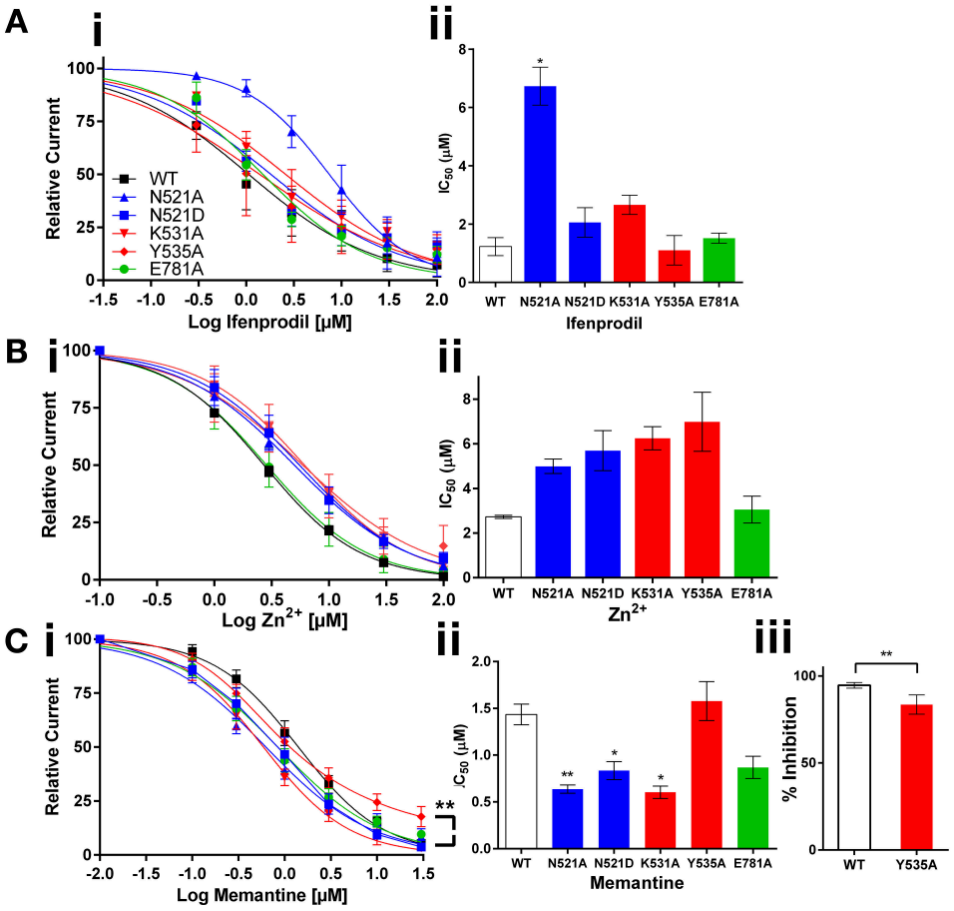
**Effect of GluN1 LBD mutations expressed with GluN2B on the activity of non-competitive antagonists**. Dose response curves and histograms show changes in potency of ifenprodil **(Ai,ii)**, and channel blockers Zn^2+^ **(Bi,ii)** and memantine **(Ci,ii)**; **C.iii** shows the % reduction in memantine efficacy with Y535A mutant. Statistical significance is marked as ^*^p < 0.01 or ^**^P < 0.001.

Ifenprodil is a GluN2B selective negative allosteric modulator binding at the interface of the GluN1/2B NTD, that is located upstream to the LBD, Figure 1A. We hypothesized that negative modulatory signals should go through the LBD to reach TMD. Therefore, the LBD mutations may have a significant effect on Ifenprodil potency. Interestingly, many mutants did not have any effect on ifenprodil activity. However, the site-I mutant (*N521A*) made ifenprodil seven-fold less potent (IC_50_:1.23 vs. 8.70 μM, *p* < 0.01) compared to the wild type receptors, Figures 2Ai,ii. These findings reveal that the sidechain of the amino acid at the GluN1 521^th^ position contributes to translating NTD mediated negative modulatory signals to the TMD.

Zn^2+^ is an endogenous antagonist that blocks the NMDA receptor with varying potency based on the GluN2 subunit combination. Zn^2+^ inhibits GluN2A with a high affinity by binding with NTD, and inhibits other GluN2 subunit containing NMDA receptors by binding only with the voltage dependent low affinity binding site at the TMD. The results obtained from the Zn^2+^ dose response curves show no significant changes in Zn^2+^ activity with the mutant receptors compared to the wild type receptors (Figures 2Bi,ii). These results fit with the logic that LBD is upstream to the channel forming TMD, and the mutations at the LBD interface may not have enough influence on the activity of Zn^2+^ that binds at the TMD. Based on the results we obtained from Zn^2+^, we anticipated LBD interactions may not have any influence on the activity of channel blockers. When considering the mechanism of channel blockers from the perspective of the binding sites along the channel axis from NTD to TMD, there is no compelling reason to anticipate any significant effect of LBD mutants on the activity of channel blockers that target the downstream TMD. However, based on the knowledge on LBD interactions in determining the dynamics of transmembrane helices, we hypothesized that LBD interactions may play role in the activity of larger (than Zn^2+^) molecules like memantine. To test this hypothesis, we have studied the effect of memantine, that binds at the extracellular vestibule of the ion channel pore by displacing endogenous Mg^2+^ ions, on mutant receptors. In agreement with our hypothesis, both site-I & II LBD mutations increased the potency of memantine [IC_50_: wt, 1.43; *N521A, 0.64 (p*< *0.001); N521D, 0.84 (p*< *0.01); K531A, 0.60* μ*M (p*< *0.01)*]. Further, the site-II mutant (*Y535A*) significantly reduced the efficacy of memantine relative to the wild type receptor (% blockade: 94.62 vs. 83.07%, *p* < 0.01, Figures 2Ciii). These results reveal that the LBD interactions are critical in determining the efficacy and potency of memantine.

## Perspective

Ligand and voltage sensitive NMDA ionotropic glutamate receptor channel function is modulated by the extracellular (NTD and LBD) domains. More than 50 x-ray crystallographic structures are available either as LBD alone or together with upstream (NTD) and downstream (TMD) domains. Historically, the LBD was considered a promising drug target because early chemical tools developed to study NMDA receptors were found to bind at the glutamate or glycine site (Monaghan et al., 1984). However, the highly conserved amino acid sequence across the NMDA receptor family of proteins reflected LBD as a less attractive target for GluN2 subunit selective inhibition or potentiation. Consequently, NTD evolved as a more attractive target for GluN2 subunit selectivity after NTD binding ifenprodil was identified (Williams, 1993) as GluN2B selective. However, ifenprodil -related compounds have remained as the only allosteric modulators capable of selectively blocking GluN2B subunit containing receptors. This scenario changed profoundly in the past 5 years, after multiple novel families of GluN2 subunit selective positive and negative allosteric modulators were identified, and evidenced LBD interface as a putative binding site (Costa et al., 2010; Mullasseril et al., 2010). These novel drug targets turned out to be of great interest that numerous high affinity GluN2A selective potentiators have been identified and co-crystallized with the LBD (Hackos et al., 2016; Volgraf et al., 2016). As LBD interface is a new drug target, in the present study we explored the role of the LBD interface in determining the potency of competitive as well as non-competitive antagonists that are not binding with this novel target.

Several trends are observable when comparing the IC_50_ (or EC_50_) values of the different compounds studied. The length of the amino acid sidechain at site I (GluN1 521st position) plays a crucial role in the interaction with the co-assembling GluN2B subunit that determines agonist potency. Increased 5,7-DCKA but unaltered DL-AP5 potency shows site-I has a relatively minor role in the overall inactivation process, which is largely controlled by the more distal part of the LBD (site-II). This is in agreement with the previous reports that site-II influences the receptor desensitization (Furukawa et al., 2005). In addition to that, the results from the present study show that *Y535A* mutation increases the potency of glycine site competitive antagonist, 5,7-DCKA. This leads to a notion that cooperative intersubunit interactions occurring in the wild type receptors positively modulate the receptor function, and attenuate the ability of glycine site antagonist to drive the receptor toward a conformation that results in channel closure.

At the TMD level, the NMDA receptor channel blocker memantine's efficacy and potency changed with the GluN1 LBD mutants. From 95% to 83% reduction efficacy does seem to be less relevant at the systemic level. However, in the native environment, presence of scaffolding proteins and their interaction with the C-terminal domains may influence the changes observed in the recombinant non-native environment in this study, warranting future exploration in the native environment, of this small but significant difference. This finding demonstrates that the positive allosteric interaction that normally exists between the GluN1/2 is not only critical for transducing NTD & LBD signals to the channel, but also refines the architecture of the transmembrane domain. Memantine is clinically used for the treatment of mild to moderate Alzheimer's disease. The results from this study support the view that minor disruption in the extracellular domain stability can increase the potency of memantine up to three-fold (~1.5–0.5 μM). In Alzheimer's disease, Aβ oligomers directly interact with the extracellular domain of NMDA receptor subunits and destabilize interdomain interactions to induce excitotoxicity (Danysz and Parsons, 2012). Thus, our findings provide a novel prediction that in AD patients memantine could bind with the malfunctioning NMDA receptors with higher potency than the ones that are functioning normally. Moreover, memantine might be the novel treatment of choice for the neurological disorders caused by the mutations in LBD of human NMDA receptor subunits, as recently identified (Yuan et al., 2015).

A number of GluN2B mutations are associated with neurological disorders including autism, intellectual disability, epilepsy, and ADHD as reviewed in (Hu et al., 2016). Also, recent findings demonstrate that development of antibodies against the GluN2B subunit is responsible for the anti-NMDAR encephalitis, and these patients suffer from clozapine refractory schizophrenia (Gon et al., 2016). On the other hand, reports demonstrate that memantine augmentation with clozapine improves the symptoms of otherwise clozapine refractory schizophrenia (Veerman et al., 2016, 2017). These studies highlight the putative role for NMDA receptors and their components in schizophrenia. As reported earlier (Gleichman et al., 2012), anti-GluN2B antibodies interact with the extracellular domains of NMDA receptors, disrupt the crucial intersubunit interactions and cause improper channel function. These patients, since their glutamate transmission that crosstalk with serotonin is imbalanced, not responding for clozapine alone treatment. These findings suggest that drugs that more specifically target sites on the NMDA receptor, and their interaction with other therapeutic drugs, may improve the treatment of schizophrenia and possibly other NMDA receptor-related psychiatric disorders. Future studies in this direction should aim to fully understand the complexity around the signal transduction mechanisms between the extracellular domains (NTD & LBD) and TMDs in helping to develop novel treatment strategies for neurological and psychiatric disorders. Furthermore, future pharmacological studies should also include behavioral assays to determine potentially beneficial vs. undesired effects of receptor manipulations.

## Closing remarks

The results from the present study demonstrate that GluN1/2B subunit LBD interactions are crucial for the normal function of the receptor. Single amino acid mutations at the GluN1 subunit LBD can disrupt the intersubunit interactions that otherwise positively modulate NMDA receptor channel function. Mutation induced negative modulatory effects were observed with competitive and non-competitive antagonists. An increase in the potency of memantine with the mutant receptors is a remarkable outcome from this study. Overall, these findings not only provide insights on pharmacology of NMDA receptor antagonists but also reinforce the perspective that LBD interactions, that positively and negatively modulate the channel, can be exploited to design and develop novel NMDA receptor based therapeutic agents.

## Author contributions

DB, CT, IM, and CM performed experiments, analyzed results and wrote the manuscript. BC designed experiments, analyzed data and wrote the manuscript. BK and BL designed experiments, analyzed data and helped with the manuscript preparation.

## Funding

This work was funded by Max Plank Society Research fellowship and One Health Grant (#10243) from the Edward Via College of Osteopathic medicine and Virginia-Maryland College of Veterinary Medicine funded to BC and BK.

### Conflict of interest statement

The authors declare that the research was conducted in the absence of any commercial or financial relationships that could be construed as a potential conflict of interest.

## Abbreviations

NMDA: N-methyl D-aspartate
LBD: Ligand Binding Domain
NTD: N-terminal Domain
TMD: Transmembrane Domain
CTD: C-terminal Domain.
